# Trace element concentrations in white leg shrimp *Litopenaeus vannamei* from retail stores in the EU, UK, and USA and the ability to discern country of origin with classification models

**DOI:** 10.1016/j.crfs.2021.09.004

**Published:** 2021-09-20

**Authors:** Robert Davis, Claude E. Boyd, Joshua Wakefield, Olga Shatova, Aaron McNevin, Blake Harris, D. Allen Davis

**Affiliations:** a Auburn University, School of Fisheries, Aquaculture, and Aquatic Sciences, 203 Swingle Hall, Auburn, AL, 36849, USA; b Oritain Global Limited, 167 High Street, Dunedin, 9016, New Zealand; c World Wildlife Fund, 1250 24th St NW, Washington, DC, 20037, USA

**Keywords:** Shrimp, Aquaculture, Sustainability, Traceability, Elemental profiling

## Abstract

Shrimp are a globally traded aquaculture commodity that accounts for a large proportion of the monetary value of aquaculture. There are concerns among consumers about seafood labeling fraud and environmental sustainability. Therefore, the geographic origin of shrimp from retail stores was investigated with trace element profiling. 94 shrimp samples were collected from grocery stores across the USA, UK, and EU in 70 different grocery stores. The results of 24 elements are reported. Shrimp samples were from Thailand, India, Vietnam, Indonesia, and Ecuador were shown to have 15 elements that were statistically different across labeled country of origin, with Ecuador having unique post hoc group membership in 5 of the elements. Based on a classification procedure, shrimp were classified to labeled country of origin with an overall accuracy of 71.2%. Overall, the results suggest that elemental profiling could be a traceability tool for classifying samples of shrimp from retail stores.

## Highlights

• Frozen Shrimp samples were obtained from grocery stores in the EU and USA.
• Samples were analyzed for element concentrations in 42 elements.
• 24 elements were detectable and presented here.
• Samples from Ecuador shown property of having a unique elemental profile.
• Samples were classified to country of origin with reasonable accuracy (71%).

## Graphical abstract

**Figure:**
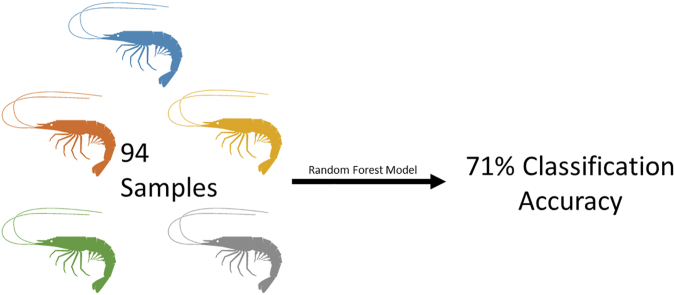

## 1 Introduction

The world's population is expected to grow to 9–10 billion by 2050 (FAO 2017). The need for animal proteins is expected to grow at an even higher rate than the population because of the growing global middle class, which will consume more meat (FAO 2009). Seafood products are an important source of nutrition for many people, and provide a source of protein, minerals, and healthy fats like Omega 3s. Currently about half of the world's seafood comes from aquaculture, and seafood accounts for about 23% of the world's meat supply (Edwards et al. 2019). Even more so than other meat products, aquaculture seafood products are global commodities where the global supply is consolidated in a few countries that send exports to international markets.

While shrimp only account for about ∼5% of the world's aquaculture production, they are disproportionally valuable as a commercial species, accounting for about 20% of the monetary value of aquaculture (FAO 2018). Shrimp production has grown dramatically in the last 15 years as the advent of specific pathogen free larvae (SPF) and better production practices have allowed for an increasingly steady supply of post larvae and higher densities in production ponds at farms. Currently, Southeast Asia is the hub of shrimp production for the world, but Ecuador is also an important source (FAO 2019). Whiteleg shrimp *Litopenaeus vannamei* is the predominant species produced, accounting for ∼83% of all penaeid shrimp aquaculture (FAO 2019). While some other shrimp species are consumed in domestic markets, whiteleg shrimp are produced almost exclusively for export, especially to developed nations like the United States (USA) and countries in the European Union (EU).

Aquatic invertebrates are well known sinks of metals, whether it be from food sources or environmental exposure (Rainbow 2002). Several factors that influence metal concentrations in shrimp tissues have been examined, including geographical variations (Li et al. 2017; Smith and Watts 2009), production source (i.e., farmed vs. wild) (Gopi et al. 2019b), and different tissues in the body (Li et al. 2014). Given that this natural variability exists in shrimp raised in differential locales, this makes shrimp from retail stores candidates for elemental profiling, which has been successful with shrimp from shrimp farms for identifying geographic origins (Li et al. 2014, 2017; Gopi et al., 2019b).

Metal concentrations in shrimp in retail markets have not been documented, and no attempts have been made with regards to identifying geographic origins in shrimp retail products based on elemental concentrations. Traceability has been a growing concern in aquaculture in recent years, and techniques to verify geographic origins have been increasingly explored in a variety of seafood items (Hassoun et al. 2020; Gopi et al. 2019a). Fraudulent labeling has been a prevalent issue in the past (Jacquet and Pauly 2008), and is likely to be a concern going forward. Therefore, the objective of this study was to document element and trace metal concentrations in whiteleg shrimp from retail stores in the USA and EU and explore the use of element concentrations in shrimp muscle tissues to verify the country of origin in whiteleg shrimp.

## 2 Methods

### 2.1 Collections

**Fig. 1:**
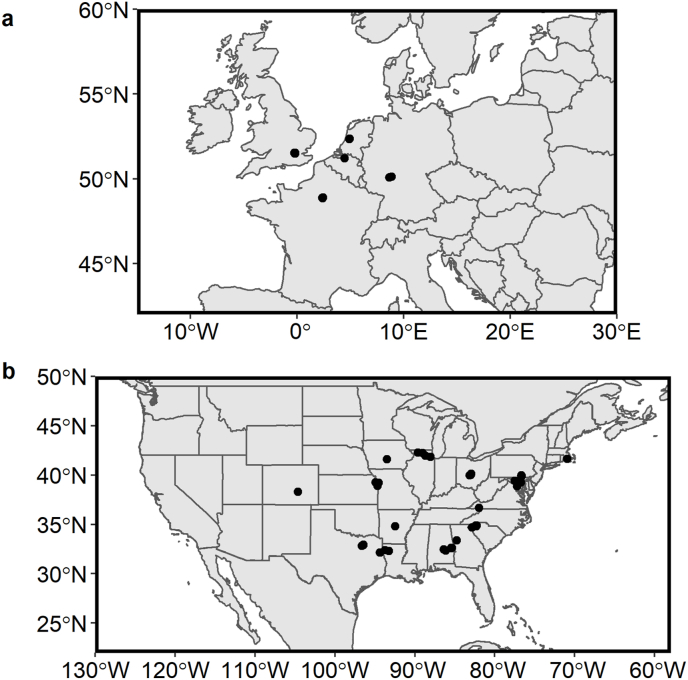
A map of sampling locations throughout the (a) Western Europe and (b) USA.

Shrimp were collected from stores in the USA and the EU between January and August of 2019. Stores were selected to cover a broad range of stores owned by unique parent companies, sampled from a range of store types (e.g., high-end organic markets, budget markets, private membership clubs, regional store chains, and national store chains). In total, 70 locations, 25 in the EU and 55 in the United States were selected as retail stores where samples were obtained (Fig. 1). Altogether, 94 samples were collected from Belgium (n = 2), France (8), Germany (6), The Netherlands (3), the United Kingdom (12), and the USA (63). Shrimp collected were from five labeled countries of origin: Ecuador (n = 12), India (30), Indonesia (21), Thailand (12), and Vietnam (19). A complete list of sampling locations and samples can be found in the supplementary information. Due to the difficulty of traveling and collecting samples, sampling locations were chosen based on a parsimonious mix of minimizing travel while maximizing the number of unique stores covered. The distribution of country of origin among samples is an artifact of the availability of shrimp from each country in the stores chosen. At each location, bags of private label and store label frozen shrimp from the supermarket's freezer section were purchased. In the USA, at least 450 g (about 1 lb) of shrimp from private brands or store brands were purchased and kept frozen until processed. Samples were chosen to be as consistent as possible with regards to size and roughly 30 count-sized shrimp were targeted. However, not all stores sold shrimp in this size so smaller and larger sizes were obtained based on availability. Shrimp were purchased in different stages of processing (whole shrimp to peeled deveined tails) but were subsequently standardized as peeled deveined tails prior to drying to maintain consistency between samples.

Shrimp were dried at 80 °C to a constant mass in the laboratory in a drying oven. In European countries, at least 1 package of shrimp (ranging from 100g to 500g) was purchased and dried with a food dehydrator (Excalibur model no. 4400220G, Excalibur Products, Sacramento CA USA) within 12 h of purchase to facilitate travel and subsequent shipping. Shrimp were dried for at least 12 h at 70 °C and stored in plastic bags dried until further processing. They were then further dehydrated to a constant mass at 80 °C in the laboratory, consistent with samples collected in the United States. Once shrimp were dried, a sub sample of three to six shrimp were ground in a IKA Economical Analytical Mill with a carbide blade (Cole-Palmer, Vernon Hills, Illinois USA) to avoid metal contamination. Dried tissue was then stored in sealed containers until digested.

### 2.2 Digestions

In preparation for digestion, samples were freeze dried overnight to remove any residual moisture. The digestion of the shrimp tissue was done following an adaptation of EPA method 200.8 (US EPA 1994) for solid materials (Environmental Express 2018). Briefly, 0.5 g of dried sample was digested with 2.0 ml of 1:1 nitric acid and 5.0 ml of 1:4 hydrochloric acid and refluxed in an Environmental Express Hotblock ^(^™^)^ (HotBlock 200, Environmental Express, Charleston SC USA) for 30 min at 85 C. Upon cooling, the samples were quantitively transferred and brought to volume in 50-ml volumetric flasks. Samples were then centrifuged at ambient temperature for 5 min and decanted to remove any insoluble material in the solution. The digestion method was verified by validating recovery of a spike quality control standard (recovery between 80 and 120%), determining the limits of detection, and repeatability of measurements with 15 replicants of a quality control standard (relative standard deviation < 20%).

### 2.3 Elemental analysis

A NexION 350d ICP-MS (PerkinElmer Inc., Waltham MA USA) was used to conduct the elemental analysis for this study. Forty-two elements were analyzed for this study: Li, B, Na, Mg, Al, K, Ca, V, Cr, Mn, Fe, Co, Ni, Cu, Zn, As, Se, Rb, Sr, Y, Mo, Ag, Cd, Sn, Sb, Cs, Ba, La, Ce, Pr, Nd, Sm, Eu, Gd, Dy, Ho, Er, Tm, Yb, Lu, Pb, U. Several steps were taken to ensure consistency between runs and within runs. The instrument used for the analysis was recalibrated with a two point calibration each day. Each run on the instrument consisted of 40 unique samples, three blanks, a lab-generated matrix matched quality control sample run in triplicate, and three other quality control materials including one replicate of a certified reference sample oyster tissue (tissue NIST1566B, MilliporeSigma, St. Louis MO USA). Two of the three quality control replicates were aqueous solutions with known quantities of each element-in the appropriate range, and the third was a salmon flesh quality control matrix-matched sample that was previously validated with recoveries between 80 and 120%, and a relative standard deviation of <20% for all elements. A small number of samples were duplicated across runs to ensure consistency between runs on the instrument. Parameters of performance in relation to the limits of detection (LoD) for each element were calculated as a blank average plus three times the standard deviation of the blanks.

### 2.4 Statistical analysis

Elements were removed from the analysis if more than 20% of the samples were below detection limits. Therefore, only the results of Al, As, B, Ba, Ca, Ce, Co, Cr, Cs, Cu, Fe, K, Li, Mg, Mn, Mo, Na, Nd, Ni, Rb, Se, Sr, Y, Zn are reported for further statistical analysis. Samples in reported elements that were below detection limits for a given element were replaced with a value at one half of the detection limit. The mean and standard deviation of element concentrations by labeled country of origin are reported. A one-way MANOVA was conducted utilizing a test statistic in Friedrich and Pauly (2018), which is robust to heteroscedasticity and can be used with high dimensional data. The reported statistic is described by Friedrich and Pauly (2018) as a “modified ANOVA type statistic” (MATS), and the p value is derived through a parametric bootstrap procedure. Following the results of the MANOVA, the mean concentrations were subsequently compared using one-way Welch's analysis of variance with a Bonferroni corrected p value (α = 0.05/24 comparisons for a significance level of α = 0.0021). Welch's ANOVA is more robust than a traditional ANOVA to heterogeneity in variation (Delacre et al. 2019). In cases where the result of the ANOVA were significant, a Games Howell pairwise comparison procedure was used to compare individual means. Games Howell pairwise comparisons are likewise more robust to heterogeneity in variation than other pairwise comparison procedures (Lee and Lee 2018). A significance level of α = 0.05 was used to determine significance in the post-hoc pairwise comparisons. Data was log transformed and subsequently centered and scaled to improve normality of the data prior to the analysis. Additionally, a principal components analysis (PCA) was conducted with elements to visualize any patterns in the underlying multivariate data structure.

Following exploration of the data with univariate tests and a PCA, two separate classification procedures were done to assess the ability to discern country of origin in samples collected from retail stores. In the first, country of origin of the retail samples was conducted with a random forest classification tree in the “caret” package in R using the method native to the “ranger” package. Recursive feature selection was used to determine the best combination of variables for the random forest. Recursive feature selection ranks the importance of variables based on their contribution to classification models and subsequently eliminates less important variables to find the most informative subsets of variables (Guyon et al. 2002). The random forest was conducted with all 24 elements following the feature selection procedure. Data was centered and scaled prior to classification to remove any effects of magnitude in the element concentrations. The importance of elements in this classification procedure were extracted and reported on a 0–100 scaling where 0 is the least important and 100 is the most important element to the classification model. The expected accuracy based on the formula in Poulin and Kamiya (2015) is reported for reference for the results of the classification procedure. The accuracy of the model was assessed with k-fold cross validation where k = 5. A second random forest classification procedure was conducted where the samples were grouped based on region of origin (Latin America vs. Asia) instead of country of origin. The recursive feature selection for the classification procedure selected seven elements (Ce, Cu, Fe, K, Mo, Nd, Y) for inclusion in the model.

To move towards the goal of country-of-origin verification, a second discrimination procedure was conducted with samples from farms collected in the countries of origin included in the retail shrimp data, which will be called the “farm data” for the purpose of this study. These samples are described in detail in Davis (2021). They will be the subject of a separate study and therefore a complete set of information about these samples will not be presented here, and they are being used to provide context for the data that is the focus of this study (i.e., the retail samples). These samples were collected following a similar procedure to that in Li et al. (2017), and subsequently classified with the same procedure as described above. There are 122, 68, 37, 48, and 53 samples from Ecuador, India, Indonesia, Thailand, and Vietnam, respectively, in the farm dataset. In this discrimination procedure, a training model was built with the farm data, and the retail samples were used as a naïve test data set. The recursive feature selection for the classification procedure for the farm data training model selected 11 elements (Al, As, Ba, Ca, Co, Cs, Li, Ni, Rb, Se, and Sr) for inclusion in the model. The random forest classification models were used because of the lack of distributional requirements for the technique, robustness to overfitting high dimensional data, the power of the model to obtain good fits when there are no strong predictor variables (Breiman 2001).

Following the classification procedure with the country of origin, Fisher's exact test were used to determine if any relationship was apparent with regards to the likelihood of correct classification with country of origin, continent where the sample was collected, and certification via aquaculture certification standards (either best aquaculture practice or aquaculture stewardship council). These factors were chosen as potential factors of interest (e.g., certification), or possible influence over the classification model (country of origin and continent from which samples were collected).

## 3 Results

### 3.1 Elemental compositions

Concentrations of individual elements in shrimp muscle tissues varied by orders of magnitude. The elements with the highest concentrations in shrimp muscle tissues from retail stores included Ca, K, Mg, and Na, which all averaged >1000 mg/kg in samples from at least one country with Na having the highest overall averages (Table 1). The elements with the lowest average concentrations out of the elements evaluated were Co, Cs, Mo, and Y with Yttrium having the lowest concentrations observed across samples from all five countries. The global MANOVA test revealed that there were differences in the shrimp muscle tissue element concentrations from different countries (MATS = 248.164, p = <0.001). After correcting for multiple comparisons, 15 elements were statistically different among the samples from the five countries: As, As, Ce, Co, Cs, Cu, Fe, K, Li, Mg, Mo, Nd, Ni, Rb, and Y. In the case of several of the elements (e.g., Al, Fe, K), shrimp muscle tissue that had a country of origin labeled as Ecuador tended to have higher element concentrations than shrimp from Asian countries. In a majority of the elements where differences were detected, Vietnam and Thailand tended belong to the same pairwise comparison groups.

**Table 1:** A summary of mean elemental concentrations in mg/kg dry weight in shrimp muscle tissues from retail stores by the labeled country of origin. The sample size from each country is listed in parentheses next to the country. The letter next the mean concentration denotes differences detected by pairwise multiple comparisons, and sd = standard deviation. The limit of detection (LOD) is listed next to each element in parentheses.

Element (LOD)	Country of Origin										
	Ecuador (12)	sd	India (30)	sd	Indonesia (21)	sd	Thailand (12)	sd	Vietnam (19)	sd	p value
**Al (0.4)**	**93.03**^**c**^	**68.855**	**28.93**^**a**^	**53.993**	**72.67**^**bc**^	**72.553**	**10.53**^**a**^	**13.277**	**37.44**^**ab**^	**60.325**	**1.08E-05**
**As (0.05)**	**1.15**^**a**^	**0.618**	**1.74**^**ab**^	**0.990**	**1.21**^**a**^	**0.547**	**2.14**^**b**^	**0.763**	**1.91**^**ab**^	**1.426**	**0.000687**
B (0.4)	1.43	0.899	1.57	1.132	2.76	2.566	1.30	0.787	1.55	1.407	0.147
Ba (0.01)	1.42	0.871	1.63	1.582	1.44	1.393	0.75	0.998	1.31	1.026	0.126
Ca (10.0)	3612.0	1707.45	2959.6	1404.05	3744.6	1284.76	2579.5	777.47	2975.5	1254.63	0.097
**Ce (0.0004)**	**0.0557**^**a**^	**0.04626**	**0.0169**^**c**^	**0.01570**	**0.0055**^**b**^	**0.00497**	**0.0061**^**b**^	**0.00599**	**0.0125**^**bc**^	**0.01529**	**3.44E-05**
**Co (0.01)**	**0.040**^**c**^	**0.0294**	**0.031**^**c**^	**0.0265**	**0.018**^**ab**^	**0.0106**	**0.009**^**a**^	**0.0056**	**0.019**^**bc**^	**0.0159**	**2.78E-05**
Cr (0.05)	0.220	0.1188	0.450	0.5954	0.513	0.7490	0.145	0.0990	0.139	0.1384	0.002
**Cs (0.0002)**	**0.014**^**c**^	**0.0090**	**0.005**^**a**^	**0.0031**	**0.009**^**ab**^	**0.0073**	**0.026**^**bc**^	**0.0125**	**0.010**^**b**^	**0.0074**	**7.48E-08**
**Cu (0.02)**	**18.57**^**b**^	**8.258**	**8.08**^**a**^	**2.217**	**7.16**^**a**^	**1.416**	**7.88**^**a**^	**2.201**	**7.47**^**a**^	**4.508**	**0.001**
**Fe (0.4)**	**76.61**^**b**^	**65.990**	**19.78**^**a**^	**14.904**	**12.33**^**a**^	**5.480**	**12.22**^**a**^	**9.955**	**17.19**^**a**^	**19.254**	**0.000247**
**K (10.0)**	**9824.1**^**b**^	**3876.10**	**6098.4**^**b**^	**2164.32**	**3091.4**^**a**^	**1567.24**	**5150.3**^**b**^	**2217.21**	**5189.0**^**b**^	**4196.79**	**1.61E-05**
**Li (0.002)**	**0.112**^**c**^	**0.0815**	**0.037**^**a**^	**0.0182**	**0.110**^**bc**^	**0.1885**	**0.054**^**ab**^	**0.0636**	**0.074**^**bc**^	**0.0329**	**1.28E-05**
**Mg (2.0)**	**1400.7**^**b**^	**225.27**	**1261.5**^**b**^	**209.84**	**951.8**^**a**^	**173.92**	**1124.4**^**ab**^	**219.55**	**1156.9**^**ab**^	**275.53**	**2.15E-05**
Mn (0.02)	1.99	0.995	1.80	1.064	2.12	1.527	0.94	0.476	1.81	0.924	0.004
**Mo (0.01)**	**0.036**^**a**^	**0.0180**	**0.116**^**b**^	**0.0964**	**0.215**^**b**^	**0.5369**	**0.103**^**ab**^	**0.1007**	**0.061**^**ab**^	**0.0605**	**4.76E-05**
Na (10.0)	22,798.0	9357.5	27,271.3	13,761.9	40,602.5	12,577.7	30,333.2	16,235.3	26,788.6	17,186.0	0.002
**Nd (0.0004)**	**0.0307**^**d**^	**0.02637**	**0.0076**^**c**^	**0.00723**	**0.0025**^**a**^	**0.00244**	**0.0025**^**ab**^	**0.00234**	**0.0065**^**bc**^	**0.00776**	**1.18E-05**
**Ni (0.02)**	**0.130**^**ab**^	**0.0668**	**0.326**^**b**^	**0.7033**	**0.201**^**ab**^	**0.2970**	**0.067**^**a**^	**0.0438**	**0.084**^**a**^	**0.0771**	**0.00071**
**Rb (0.005)**	**2.76**^**b**^	**1.308**	**1.75**^**b**^	**0.820**	**0.88**^**a**^	**0.377**	**1.80**^**b**^	**0.723**	**1.45**^**ab**^	**0.991**	**1.12E-05**
Se (0.05)	1.22	0.250	1.28	0.377	1.17	0.262	1.20	0.372	1.15	0.163	0.765
Sr (0.004)	42.63	21.3117	28.70	15.0207	45.38	17.3434	25.44	9.5675	28.49	13.6779	0.002
**Y (0.001)**	**0.031**^**b**^	**0.0242**	**0.008**^**b**^	**0.0069**	**0.009**^**b**^	**0.0230**	**0.003**^**a**^	**0.0025**	**0.008**^**b**^	**0.0095**	**0.000876**
Zn (0.4)	48.95	2.7324	48.83	4.8461	45.57	4.1944	47.15	3.4685	47.28	5.7204	0.07

**Fig. 2:**
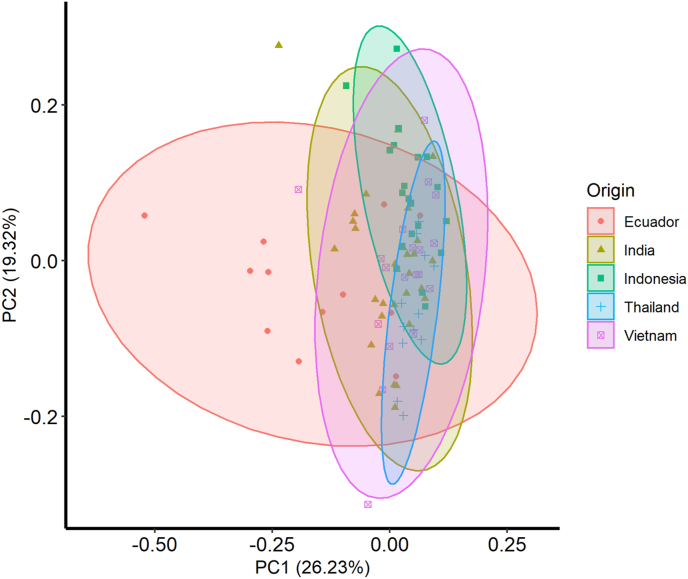
A bi-plot of the first and second component of the principal components analysis. The variation explained in the data is in parentheses. Samples from different countries are represented by different shape color/combinations. The combinations are; i. red circles = Ecuador, ii. yellow triangles = India, iii. Green diamonds = Indonesia, iv. Blue crosses = Thailand, and v. purple boxes = Vietnam. Ellipses represent normalized group ellipses in two-dimensional space. (For interpretation of the references to color in this figure legend, the reader is referred to the Web version of this article.)

A PCA was conducted to help discern any underlying patterns in the data that may be useful in terms of classification. The first two principal components are plotted in Fig. 2, which capture 26.23% and 19.33% of the variation in the data, respectively. The shrimp samples from Ecuador appear to separating across the first principal component, which is most strongly associated with Ce, Co, Cu, Fe, and Y (see Table 2). The four Asian countries in the dataset do not appear to have distinct groups but are primarily spread across the second principal component which is most strongly associated with Al, As, K, Mg, and Mn.

**Table 2:** Eigenvectors, eigenvalues, and variation explained of the data in this study. Principal components with an eigenvalue >1 are presented.

Element	PC1	PC2	PC3	PC4	PC5	PC6
Al	−0.14	0.24	−0.21	−0.22	0.15	0.23
As	−0.02	−0.30	0.03	0.14	0.10	0.03
B	0.03	0.12	−0.34	0.27	0.18	−0.35
Ba	−0.12	0.22	0.30	0.00	−0.13	0.00
Ca	−0.16	0.22	0.09	0.34	−0.47	0.15
Ce	−0.37	0.04	−0.11	−0.17	−0.01	−0.08
Co	−0.31	0.15	0.17	0.02	0.23	−0.11
Cr	−0.08	0.23	0.30	0.23	0.37	0.15
Cs	−0.10	−0.20	−0.24	0.32	0.06	0.11
Cu	−0.31	−0.14	−0.09	0.05	0.01	0.13
Fe	−0.36	0.05	−0.16	−0.13	0.05	−0.04
K	−0.23	−0.29	−0.02	0.06	0.09	0.17
Li	−0.08	0.06	−0.30	0.22	−0.03	−0.43
Mg	−0.21	−0.30	0.13	0.16	−0.10	−0.05
Mn	−0.17	0.28	0.12	−0.10	0.05	−0.08
Mo	0.02	0.04	−0.13	0.45	0.18	−0.21
Na	0.10	0.31	−0.26	0.03	0.07	0.25
Nd	−0.37	0.03	−0.14	−0.17	−0.01	−0.06
Ni	−0.11	0.20	0.33	0.23	0.41	0.05
Rb	−0.23	−0.31	0.01	0.07	0.14	0.19
Se	−0.02	0.03	0.22	−0.18	−0.03	−0.55
Sr	−0.14	0.23	0.03	0.34	−0.47	0.11
Y	−0.31	0.11	−0.14	−0.15	−0.12	−0.05
Zn	−0.12	−0.24	0.33	0.05	−0.13	−0.22
Eigenvalue	2.51	2.15	1.64	1.26	1.26	1.14
Percent Variation	26.23	19.33	11.16	6.652	6.582	5.424
Cumulative	26.23	45.56	56.72	63.372	69.954	75.378

### 3.2 Classification

**Fig. 3:**
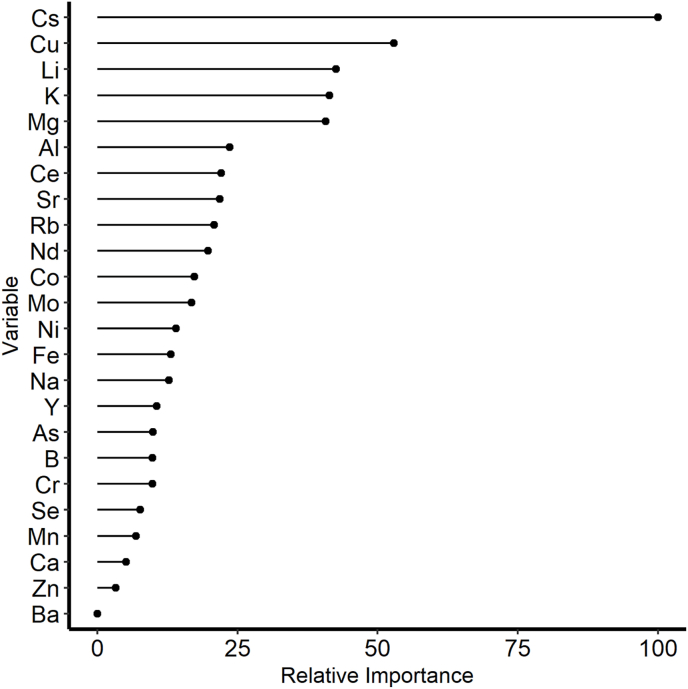
The relative importance of the elements in the classification of the retail samples with a random forest discriminant model. The importance of the variables is scaled so that the most important is given a score of 100 and the least important is assigned a score of zero.

Overall, the random forest classification of the retail samples successfully classified 71.2% of the samples into their respective country of origin based on K-fold cross validation (Table 3). The expected accuracy based on random chance for this dataset was 22.5% and Cohen's kappa for this test was 0.62 (std. error = 0.061) (Cohen, 1960). The most important elements to the classification model were Cs, Cu, Li, K, and Mg with a drop off to the rest of the elements, decreasing to Ba, which was the least important element (Fig. 3). The country with the highest rate of success was India, where 93% of the samples were correctly identified as being from India by the model. Conversely, the lowest country level classification was Vietnam, which was also frequently classified as being from India. When the Asian countries are collapsed into a single region classifier, the resulting classifications would be accurate 94.7% of the time (8/12 in Ecuador and 81/82 in Asia). In both cases, four samples from Ecuador were incorrectly classified as being from Asia, specifically India (3) and Indonesia (1). A second procedure was conducted where samples collected from shrimp farms were used as a training set and the samples of interest here (the “retail samples”), were used as a naïve test set. The results of the training model are presented in the supplementary information, Table 1. The baseline model with the farm samples achieved an accuracy of 90% based on k-fold cross validation, while the classification of the identity of the retail samples with the farm data model only achieved an overall accuracy of 40% (Table 4). The country that performed most poorly in the retail samples as a naïve test set was Indonesia, where only about 10% of the samples were correctly classified. The country with the best performance in the naïve test set was Ecuador, where 67% of the samples were correctly classified. Cohens Kappa for the naïve test was 0.259 (std. error = 0.0258).

**Table 3:** The classification of samples in this study to country of origin with a random forest model. The samples are assigned a classification based k-fold cross validation.

	Reference					
Prediction	Ecuador	India	Indonesia	Thailand	Vietnam	
Ecuador	8	0	0	0	1	
India	3	28	2	0	6	
Indonesia	1	2	16	2	3	
Thailand	0	0	2	8	2	
Vietnam	0	0	1	2	7	
Accuracy	67%	93%	76%	66%	37%	
Overall						71.20%

**Table 4:** The results of the retail samples as naïve testing data when classified against the farm samples as training data.

Prediction	Reference					
	Ecuador	India	Indonesia	Thailand	Vietnam	
Ecuador	8	7	14	1	7	
India	0	18	3	1	3	
Indonesia	3	4	2	2	1	
Thailand	1	0	2	6	4	
Vietnam	0	1	0	2	4	
Accuracy	67%	60%	9.5%	50%	21%	
Overall						40.4%

The relationship between external factors and classification success were subsequently examined with Fisher exact tests to determine if important factors (e.g., certification) were related to the success of sample classification. Altogether, 48 out of the 94 samples had certifications with the most common being BAP (n = 30) and ASC (n = 13), with the rest of the assorted certified samples being others. There was no relationship detected between classification success and the location the samples were obtained, be it the USA or Europe (*p =* 0.063). No relationship was detected between certification status of the samples and classification success (*p* = 0.1311). A relationship was determined to exist between the country of origin of the sample and the success of classification (*p* = 0.009).

## 4 Discussion

Shrimp are an important seafood commodity on the global market, accounting for approximately 20% of the total value of aquaculture globally (FAO 2018). However, production is consolidated into a few countries, especially that of the most widely traded species whiteleg shrimp, and therefore a robust international trade exists for aquaculture shrimp. Seafood labeling fraud has been widely documented in importing markets such as Europe (Christiansen et al. 2018; Jacquet and Pauly 2008) and the USA (Lagasse et al. 2014; Korzik et al. 2020), and given environmental problems of the past in shrimp aquaculture (Naylor et al. 2000; Bailey 1988; Richards and Friess 2016; Holmstrom et al. 2003) and recent allegations of human rights violations (Hodal et al. 2014), there is a growing interest in improving the traceability of seafood products. Elemental profiling is a tool that has been proposed to delineate a pre-determined groups (Hassoun et al., 2020; Gopi et al., 2019a; Davis et al. 2021) and therefore improve traceability. Here, we explore the potential to delineate the country of origin of retail shrimp products based on the element concentrations in the tail muscle tissues, the first attempt of its kind in retail shrimp products.

Elemental profiling has proliferated as a method to delineate geographic origins in shrimp over the last two decades and has been highly successful. Smith and Watts (2009) were able to discern shrimp samples from 8 different countries with >70% accuracy in their discrimination procedure. This dataset and methodology was developed as part of a customs and border patrol case where several shipments of shrimp products from Charoen Pokphand's CP PRIMA in Indonesia were seized out of Indonesia by the US Customs and Border Patrol (US Customs and Border Patrol 2010). These samples were believed to be transshipped, meaning they were grown in a different country and sent to Indonesia for packaging (Kohn Ross 2005), however the samples were later released. Li et al. (2017) were able to classify shrimp to three Asian countries with over a 97% overall accuracy, and Gopi et al. (2019b) were able to obtain >82% accuracy with shrimp from five countries. Here, the random forest obtained a 71.2% overall accuracy, which is the same or lower than the examples discussed above, and lower than several other examples with shrimp (Davis et al., 2021).

Extenuating factors may have played a role in the relatively low classification accuracy of 71.2% in this study. Several factors have been shown to affect element concentrations in shrimp tissue. Besides geography, the overall size of the shrimp plays a role in the elemental concentrations in the tissues (Boyd and Teichert-Coddington 1995). Boyd and Teichert-Coddington (1995) show that whiteleg shrimp across a range of sizes from (1.7 g–24.2 g) have slightly different mineral compositions, which could be enough to affect elemental profiling. In this study, attempts were made to obtain shrimp of similar sizes, however this was based upon availability of shrimp at the stores where shrimp were purchased and did vary to some degree (see supplemental information). Additionally, because of the role in metallothionein with metal movement in shrimp tissues when frozen (Pourang et al. 2004, 2005), and chemical treatments at the pond and in post-harvest processing with chemicals like sodium metabisulfite, sodium chloride, and polyphosphates (Boyd and McNevin 2014), there is reason to suspect that metal concentrations in shrimps post-harvest could be different than shrimp obtained from farms in countries where they are grown. As an example, the Na concentrations in this study are approximately five times what is reported in Li et al. (2017) and in the farm data in this study in shrimp from the same countries. This may present a challenge in any future attempts to use shrimp captured from farms as a validated database from which to identify country of origin in shrimp retail products. The validation of country of origin with the farm samples with the retail samples as a naïve test set here was basically unsuccessful and past attempts by the authors (unpublished data) have likewise yielded middling results. Another farm level factor that could play a role is the salinity of the ponds, which has been demonstrated to be a factor that can be distinguished via elemental profiling (Li et al. 2019). Due to the nature of the sampling here, it is unknown what the salinity of the water in which the shrimp were reared and to what extent that is affecting the results. However, Li et al. (2014) was relatively unsuccessful in correlating water elemental concentrations with tissue levels and past attempts of elemental profiling that were highly successful (Li et al., 2017) would have captured variation in salinity levels in ponds.

Analytical differences in the data could potentially cause small differences, however the elemental concentrations here for retail shrimp are in good agreement with the USDA's food data for shrimp (USDA 2020), so it is unlikely that the analytical procedure is playing a significant role in the differences. A final confounding factor is the possibility that some samples in the data were transshipped, and therefore not labeled with the correct country of origin. It is difficult to ascertain the extent this could be a factor in the accuracy of the classification model.

The patterns in the elemental concentrations suggest that there are distinct differences in some of the countries in this study that will lend themselves to accurate elemental profiles in future efforts. Some elements were shown to have distinct group membership via pairwise comparisons for certain countries (e.g., Ecuador in Fe and Nd, Indonesia in K). This is also partially confirmed by the relative importance of the elements in the classification model, where Cs is the most important element and has three countries that are unique based on post hoc pairwise comparisons (Ecuador, Vietnam, and India). The next four most important elements, Cu, Li, K, and Mg also have at least one country that is statistically different than the other countries. Ecuador specifically was different with at least 3 of the 4 Asian countries in 5 out of the 15 elements where statistical differences were detected, suggesting that samples from Ecuador have a unique profile. With a greater sample size, it is likely this would lead to higher levels of correct classification in the discriminant models; however, it was difficult to obtain samples of Ecuadorian shrimp in retail markets. All twelve of the samples in this study that had Ecuador as a country of origin were from the EU. On the contrary, the data in this study supports previous research that shows samples from Thailand and Vietnam are more difficult to discriminate from each other than they are with other countries. In Li et al. (2017), attempts to classify samples to regions within Vietnam and Thailand were not as successful as attempts to discriminate these countries from other countries. In this study, Thailand and Vietnam were only statistically different from one another in the element Co, while they were statistically the same in the 11 other elements where there was statistical difference.

## 5 Conclusions

Elemental profiling is a well-researched tool that has been proposed as a means to identify the geographic origins of seafood products. This is the first attempt to identify retail shrimp products to country of origin based on elemental profiling, and this study highlights some of the challenges that profiling retail samples presents. These results are preliminary and suggest that with a large robust sample, it may be possible to identify the country of origin in retail products independent of their labeling. Based on the observation that some elements may be different in post-processed shrimp obtained from retail stores when compared to samples collected from farms, a database based on post-processing plant samples may be more desirable for the validation of retail sample when compared to samples obtained from farm ponds, which could overcome some of the difficulties observed with profiling samples from retail stores. Additionally, shrimp shells may be more resilient to industrial processing, and should be explored as an alternative tissue to shrimp muscle tissue. Overall, there is potential to apply this technique to seafood retail products.

## CRediT authorship contribution statement

**Robert Davis:** Conceptualization, Methodology, Formal analysis, Data curation, Writing – original draft, Visualization. **Claude E. Boyd:** Conceptualization, Writing – original draft, Writing – review & editing, Supervision, Funding acquisition. **Joshua Wakefield:** Methodology, Formal analysis, Writing – review & editing. **Olga Shatova:** Methodology, Formal analysis, Writing – review & editing. **Aaron McNevin:** Conceptualization, Funding acquisition, Writing – review & editing. **Blake Harris:** Conceptualization, Funding acquisition, Writing – review & editing. **D. Allen Davis:** Conceptualization, Writing – original draft, Writing – review & editing, Supervision, Funding acquisition.

## Declaration of competing interest

The authors declare that they have no known competing financial interests or personal relationships that could have appeared to influence the work reported in this paper.
