# Supplementary material for: Trace element concentrations in white leg shrimp Litopenaeus vannamei from retail stores in the EU, UK, and USA and the ability to discern country of origin with classification models
Source: Curr Res Food Sci. 2021 Sep 20;4:655–61. doi: 10.1016/j.crfs.2021.09.004 (PMC8473577; doi:10.1016/j.crfs.2021.09.004)
Supplement: Multimedia component 1 [file mmc1.docx]

SI Table 1. A complete list of sampling locations and brands purchased.

| Sample | Grocery Store | Brand | Country | Lat | Long | Size | Labeled Country of Origin | Certification |
| --- | --- | --- | --- | --- | --- | --- | --- | --- |
| 1 | Giant | Nature Promise (Giant Organic) | USA | 39.91854 | -76.628 | 31-40 CT | India | None |
| 2 | Giant | Giant Brand | USA | 39.91854 | -76.628 | 51-60 CT | Thailand | None |
| 3 | Save-a-Lot | Captain Bob's | USA | 39.96116 | -76.7038 | 40-60 CT | India | HACCP |
| 4 | Sam's Club | Membersmark | USA | 39.9803 | -76.6743 | 50-70 CT | India | BAP 2 Star |
| 5 | Nell's Market | Wholey | USA | 39.90687 | -76.6809 | 26-30 CT | Vietnam | HACCP |
| 6 | Nell's Market | Best Yet | USA | 39.90687 | -76.6809 | 31-40 CT | Indonesia | HACCP |
| 7 | Price Rite | Tastee Choice | USA | 39.93462 | -76.6932 | 31-40 CT | India | BAP 2 Star |
| 8 | Price Rite | Cape Gourmet | USA | 39.93462 | -76.6932 | 31-40 CT | Indonesia | BAP 2 Star |
| 9 | Grocery Outlet | Ocean Gift | USA | 39.95543 | -76.6815 | 26-30 CT | Indonesia | BAP 2 Star |
| 10 | Grocery Outlet | Chicken of the Sea | USA | 39.95543 | -76.6815 | 16-20 CT | Indonesia | BAP 1 Star |
| 11 | Wegmans | Ultra | USA | 39.41345 | -76.7747 | 21-25 CT | Thailand | BAP 3 Star |
| 12 | BJ's | Wellsley Farms | USA | 39.40722 | -76.7761 | 21-25 CT | India | None |
| 13 | Safeway | Waterfront Bistro | USA | 39.43875 | -77.5253 | 31-40 CT | Indonesia | None |
| 14 | Giant Eagle | Sail | USA | 39.43127 | -77.4188 | 26-30 CT | Indonesia | None |
| 15 | Giant Eagle | SeaMazz | USA | 39.43127 | -77.4188 | 31-40 CT | Indonesia | BAP 2 Star |
| 16 | Giant Eagle | Nature's Basket | USA | 39.43127 | -77.4188 | 26-30 CT | India | None |
| 17 | Moms Organic Market | BMR | USA | 38.87346 | -77.231 | 16-20 CT | India | None |
| 18 | Food City | Cape Covell | USA | 36.70133 | -81.9774 | 41-50 CT | Indonesia | None |
| 19 | Lowes Foods | Black Tie | USA | 34.90114 | -82.2545 | 26-30 CT | Thailand | HACCP, BAP 1 Star, "Turtle Safe" |
| 20 | Bi Lo | Fishermans Wharf | USA | 34.89499 | -82.2898 | 16-20 CT | Indonesia | BAP 4 Star |
| 21 | Ingles | Seabest | USA | 34.9039 | -82.3492 | 26-30 CT | India | BAP 2 Star |
| 22 | Lidl | No Branding (Store Brand) | USA | 34.88501 | -82.3553 | 31-40 CT | Thailand | BAP 2 Star |
| 23 | Trader Joes | Trader Joes | USA | 34.82933 | -82.3059 | 21-30 CT | Thailand | None |
| 24 | Aldi | Fremont Fish Market | USA | 32.46146 | -86.4207 | 41-60 CT | India | BAP 4 Star |
| 25 | Whole Foods | "Farmed Raised Shrimp" | USA | 32.35861 | -86.1725 | 51-60 CT | Vietnam | "Third Party Cert |
| 26 | Costco | Kirklands | USA | 32.36283 | -86.1507 | 50-70 CT | India | None |
| 27 | Target | Market Pantry | USA | 32.61761 | -85.4105 | 41-50 CT | Indonesia | BAP 2 Star |
| 28 | Kroger | Kroger | USA | 32.62086 | -85.4113 | Large | Indonesia | BAP 2 Star |
| 29 | Piggly Wiggly | Natures Best | USA | 32.64155 | -85.391 | 41-50 CT | India | None |
| 30 | Walmart | Captains Pack | USA | 32.63761 | -85.4219 | 31-40 CT | India | BAP 4 Star |
| 31 | Publix | Publix | USA | 32.63789 | -85.4802 | 31-35 CT | Indonesia | None |
| 32 | Publix | Chicken of the Sea | USA | 32.63789 | -85.4802 | 13-15 CT | Thailand | BAP 2 Star |
| 33 | Albertsons | Waterfront Bistro | USA | 38.31065 | -104.629 | 31-40 CT | Indonesia | None |
| 34 | Albertsons | Waterfront Bistro | USA | 38.31307 | -104.629 | 26-30 CT | Indonesia | None |
| 35 | Schnucks | KNC Agro Lmtd | USA | 42.22803 | -89.069 | 16-20 CT | India | None |
| 36 | Hyvee | Hyvee Fish Market | USA | 41.95903 | -88.7192 | 26-30 CT | Thailand | BAP 2 Star |
| 37 | Target | Market Pantry | USA | 38.90818 | -94.6748 | 41-50 CT | India | BAP 2 Star |
| 38 | Jewel-Osco | Waterfront Bistro | USA | 41.85874 | -88.0627 | 26-30 CT | Indonesia | None |
| 39 | Hyvee | Hyvee Fish Market | USA | 39.22342 | -94.547 | 26-30 CT | Thailand | BAP 2 Star |
| 40 | Tom Thumb | Waterfront Bistro | USA | 32.96444 | -96.4629 | 26-30 CT | Indonesia | None |
| 41 | Costco | Kirklands | USA | 32.9081 | -96.4461 | 21-25 CT | India | None |
| 42 | Fareway Foods | Supreme Choice | USA | 41.60022 | -93.5055 | 31-40 CT | Indonesia | None |
| 43 | Dillons | Kroger | USA | 39.26763 | -94.92 | extra large' | India | None |
| 44 | Cubs | Artic Shores | USA | 42.28325 | -89.6326 | 26-30 CT | Indonesia | BAP 2 Star |
| 45 | Winco | Winco | USA | 32.85515 | -96.6443 | 41-50 CT | Vietnam | BAP 4 Star |
| 46 | Price Chopper | Aquastar | USA | 39.21124 | -94.5469 | 31-40 CT | Indonesia | BAP 2 Star |
| 47 | Walmart | Walmart Brand | USA | 34.80973 | -92.4867 | 26-30 CT | India | BAP 4 Star |
| 48 | Brookshire Brothers | Brookshire Brothers | USA | 32.15713 | -94.3452 | 51-60 CT | India | None |
| 49 | Brookshire | Full Cirlce | USA | 32.39966 | -93.763 | 21-25 CT | India | None |
| 50 | Supervalu | Diamond Reef | USA | 32.32831 | -93.2795 | 31-40 CT | India | None |
| 51 | Aldi | Aldi Fremont Market | USA | 32.96618 | -96.4649 | 41-60 CT | India | BAP 4 Star |
| 52 | H-E-B | Great Catch | USA | 32.15924 | -94.3724 | 26-30 CT | Indonesia | None |
| 53 | Publix | Publix | USA | 33.3958 | -84.7638 | 41-50 CT | India | None |
| 54 | Giant Eagle | Giant Eagle | USA | 40.01942 | -83.0131 | 26-30 CT | India | BAP 4 Star |
| 55 | Food Lion | Food Lion | USA | 34.69661 | -82.8774 | 31-40 CT | India | BAP 2 Star |
| 56 | Lucky's | Lucky Brand | USA | 40.01885 | -83.0116 | 26-30 CT | India | None |
| 57 | Food Lion | Oishi | USA | 34.69661 | -82.8774 | 13-15 CT | Thailand | None |
| 58 | Kroger | Simple Truth | USA | 40.03198 | -83.0173 | "Jumbo" | Thailand | None |
| 59 | Meijer | Meijer Brand | USA | 39.98391 | -83.1536 | 26-30 CT | India | BAP 4 Star |
| 60 | Costco | Kirklands | USA | 40.15083 | -82.9767 | 31-40 CT | India | None |
| 61 | HMart | Tong Tong Bay | USA | 39.28235 | -76.7548 | 26-30 CT | Thailand | None |
| 62 | HMart | Fresh | USA | 39.28235 | -76.7548 | 26-30 CT | Indonesia | None |
| 63 | Market Basket | Market Basket | USA | 41.65747 | -70.923 | 26-30 CT | India | BAP |
| 64 | Iceland | Artic Royal | United Kingdom | 51.53912 | -0.19456 | N/A | India | None |
| 65 | Blue Ocean | Wharf | United Kingdom | 51.50671 | -0.26876 | N/A | Ecuador | None |
| 66 | Iceland | Iceland | United Kingdom | 51.53912 | -0.19456 | N/A | Vietnam | None |
| 67 | Sainsbury | Sainbury | United Kingdom | 51.53871 | -0.19414 | N/A | Vietnam | None |
| 68 | ASDA | ASDA | United Kingdom | 51.5289 | -0.26981 | N/A | Vietnam | None |
| 69 | ALDI | ALDI | United Kingdom | 51.53944 | -0.19517 | N/A | Vietnam | BAP 4 Star |
| 70 | Ranya | Aquahawk | United Kingdom | 51.5402 | -0.196 | N/A | Vietnam | BAP 1 Star |
| 71 | Morrisons | Morrisons | United Kingdom | 52.50827 | -0.27255 | N/A | Vietnam | None |
| 72 | Coop | Coop | United Kingdom | 51.4925 | -0.19362 | N/A | Vietnam | None |
| 73 | LIDL | LIDL | United Kingdom | 51.50294 | -0.21837 | N/A | Ecuador | ASC |
| 74 | Waitrose | Waitrose | United Kingdom | 51.50634 | -0.2196 | N/A | Vietnam | None |
| 75 | Tesco | Tesco | United Kingdom | 51.50663 | -0.249 | N/A | Vietnam | None |
| 76 | Auchan | Delpierre | France | 48.87851 | 2.385617 | N/A | Ecuador | ASC |
| 77 | Monoprix | Monoprix | France | 48.86625 | 2.361366 | N/A | Ecuador | None |
| 78 | Carrefour | Carrefour Bio | France | 48.86165 | 2.367758 | N/A | Ecuador | AB Certificate? |
| 79 | Intermarche | Pescanova | France | 48.86825 | 2.359963 | N/A | Ecuador | ASC |
| 80 | Carrefour | Maison Mar | France | 48.86165 | 2.367758 | N/A | Ecuador | ASC |
| 81 | E. Leclerc | Crevettes | France | 48.89137 | 2.404411 | N/A | Ecuador | None |
| 82 | E. Leclerc | Tinu's | France | 48.89137 | 2.404411 | N/A | Ecuador | None |
| 83 | Super U | U Bio | France | 48.86461 | 2.372461 | N/A | Ecuador | None |
| 84 | Colruyt | Colruyt | Belgium | 51.21061 | 4.455744 | N/A | Vietnam | ASC |
| 85 | LIDL | Ocean Sea | Belgium | 51.21038 | 4.455009 | N/A | Thailand | ASC |
| 86 | Spar | Bio+ | Netherlands | 52.36417 | 4.905266 | N/A | Ecuador | None |
| 87 | Albert Heijn | Albert Heijn | Netherlands | 52.35771 | 4.89642 | N/A | Vietnam | None |
| 88 | Albert Heijn | Del Mare | Netherlands | 52.35771 | 4.89642 | N/A | Vietnam | ASC |
| 89 | Jumbo | Jumbo | Netherlands | 52.35777 | 4.895896 | N/A | Vietnam | ASC |
| 90 | Edeka | Edeka | Germany | 50.08172 | 8.634147 | N/A | Vietnam | ASC |
| 91 | Tegut… | Tegut… | Germany | 50.10505 | 8.685015 | N/A | India | ASC |
| 92 | Globus | Escal | Germany | 50.13036 | 8.86762 | N/A | Ecuador | ASC |
| 93 | Rewe | White Tiger Fish and More | Germany | 50.10166 | 8.686788 | N/A | Vietnam | ASC |
| 94 | Aldi | Alta | Germany | 50.10321 | 8.690295 | N/A | Vietnam | ASC |
